# Propofol Suppresses Microglia Inflammation by Targeting TGM2/NF-*κ*B Signaling

**DOI:** 10.1155/2021/4754454

**Published:** 2021-08-24

**Authors:** Yuanyuan Hou, Xi Xiao, Wei Yu, Sihua Qi

**Affiliations:** ^1^Department of Anesthesiology, The Fourth Affiliated Hospital of the Harbin Medical University, Harbin, 150001 Heilongjiang Province, China; ^2^Department of Anesthesiology, First Affiliated Hospital of Dalian Medical University, Dalian, 116011 Liaoning Province, China

## Abstract

**Background:**

Propofol is a known intravenous hypnotic drug used for induction and maintenance of sedation and general anesthesia. Emerging studies also reveal a neuroprotective effect of propofol in diverse diseases of neuronal injuries via modulating microglia activation. In this study, we aimed to uncover the downstream targets of propofol in this process.

**Methods:**

RNA sequencing analysis to identify genes implicated in the propofol-mediated neuroprotective effect. Quantitative real-time PCR, enzyme-linked immunosorbent assay, and Western blotting analysis were performed to analyze inflammatory gene expression, cytokine levels, and TGM2. BV2 cells and primary microglia were used for functional verification and mechanism studies.

**Results:**

The multifunctional enzyme transglutaminase 2 (TGM2) was identified as a putative functional mediator of propofol. TGM2 was significantly upregulated in lipopolysaccharide- (LPS-) primed BV2 cells. Genetic silencing of TGM2 abolished LPS-induced microglial activation. Notably, gain-of-function experiments showed that the proinflammatory effects of TGM2 were dependent on its GTP binding activity instead of transamidase activity. Then, TGM2 was revealed to activate the NF-*κ*B signaling pathway to facilitate microglial activation. Propofol can inhibit TGM2 expression and NF-*κ*B signaling in BV2 cells and primary microglia. Ectopic expression of TGM2 or constitutively active IKK*β* (CA-IKK*β*) can compromise propofol-induced anti-inflammatory effects.

**Conclusions:**

Our findings suggest that TGM2-mediated activation of NF-*κ*B signaling is an important mechanism in the propofol-induced neuroprotective effect that prevents microglial activation.

## 1. Introduction

Accumulated studies have revealed that general anesthetics could induce neurotoxicity, followed by long-lasting memory and learning abnormalities. Propofol (2,6-diisopropylphenol) is a commonly used intravenous anesthetic agent not only for the induction and maintenance of anesthesia but also in the intensive care unit (ICU) [1, 2]. Different from other anesthetic drugs, multiple animal models showed that propofol might exhibit a neuroprotective effect in neuronal injuries such as ischemia reperfusion injury and traumatic brain injury [3–5]. Mechanistically, propofol has been found to suppress cell apoptosis and inflammation and to influence neuroprotection-associated proteins to exert its neuroprotective effects [6]. Neuroinflammation is a multifaceted response in the central nervous system (CNS). Microglia are deemed as crucial players in initiating an inflammatory response after injury. Microglia can secrete various inflammatory cytokines, chemokines, and growth factors and undergo significant morphological changes in response to CNS injuries [7–9]. Propofol has been reported to exert several anti-inflammatory effects on microglia. However, knowledge regarding the molecular mechanism underlying the anti-inflammatory properties of propofol in microglia activation is still limited.

Transglutaminase 2 (TGM2) is a family member of the transglutaminase (TGM) enzymes, which contain 8 kinds of discrepant enzyme subtypes designated as blood coagulation factor XIII and TGM1-7 [10]. TGM2, also known as tissue transglutaminase, is constitutively expressed in various organ-specific cells such as smooth muscle cells and fibroblasts [11]. Apart from its known Ca^2+^-dependent protein crosslinking activity, TGM2 also plays diverse roles, including but not limited to hydrolyzing ATP and GTP to mediate signal transduction through G-protein-coupled receptors (GPCRs), protein disulfide isomerase activity, protein kinase activity, and interacting with several proteins as a molecular scaffold [12, 13]. These molecular functions of TGM2 participate in the regulation of diverse pathophysiological processes such as wound healing, cell growth, cell differentiation, cell apoptosis, extracellular microenvironment, autophagy, and tumor initiation and progression [14–18]. TGM2 is implicated in modulating glial cell function such as astrocytes, microglia, and oligodendrocytes and contributes to pathophysiology in the central nervous system (CNS) [19]. Previously, it has been reported that an increase of TGM2 expression facilitates the generation of inducible nitric oxide synthase (iNOS), which might induce neuronal death [20]. Therefore, controlling TGM2 activity might provide an approach to protect neurons and care for CNS injuries [21].

In the present study, we identified the molecular mechanisms underlying the protective effects of propofol in LPS-induced neuroinflammation. To achieve this, we analyzed genes upregulated by LPS stimulation and genes downregulated by propofol treatment. Here, we revealed that activated microglia highly expressed TGM2, which contributed to microglial activation via activating of NF-*κ*B signaling. Moreover, using loss- and gain-of-function experiments, we confirmed the roles of the TGM2-NF-*κ*B axis in the anti-inflammatory function of propofol in microglia activation.

## 2. Materials and Methods

### 2.1. Cell Culture and Reagents

The BV2 cell line was acquired from the China Center for Type Culture Collection (Wuhan, China). BV2 cells were cultured in Dulbecco's modified Eagle's medium (Gibco, Shanghai, China) supplemented with 10% (*v*/*v*) fetal bovine serum (FBS, Gibco, Shanghai, China), 100 U/ml penicillin, and 100 mg/ml streptomycin (Life Technologies, USA). BV2 cells were grown in a humidified atmosphere containing 5% CO_2_ at 37°C. Propofol and lipopolysaccharide (LPS) were all obtained from Sigma-Aldrich (St. Louis, MO, USA) and dissolved in DMSO to prepare a stock solution. GK921 was purchased from Selleck (S6417, Shanghai, China).

### 2.2. Cell Transfection

Small interfering RNAs (siRNAs) against *Tgm2* and its negative control were obtained from GenePharma, Inc. (Shanghai, China). The detailed sequences of siRNAs against *Tgm2* were shown as follows: si-*Tgm2*-1, 5′-GGCAGAAGAUCAGACUAATT-3′; si-*Tgm2*-2, 5′-GCCUGAUGCUCUUGGAUAUAUTT-3′; and si-Ctrl, 5′-UUCUCCGAACGUGUCACGUTT-3′. Transient transfection was performed using the jetPRIME transfection reagent (114-15, Polyplus-transfection, France) according to the manufacturer's instructions. The knockdown efficiency of TGM2 in BV2 cells was evaluated by Western blotting. Constructs for mouse wide-type TGM2, transamidase-inactive TGM2 (C277S), and GTP-binding-inactive TG2 (R580A) were generated by GenePharma, Inc. (Shanghai, China) and cloned to the pcDNA3.1 plasmids for transfection. Transfection of these plasmids was performed with the Lipofectamine 2000 reagent (#11668-027, Invitrogen, USA) according to the manufacturer's protocol.

### 2.3. RNA Sequencing Experiment

RNA sequencing (RNA-seq) analysis was carried out to decipher the molecular changes induced by LPS in the presence or absence of propofol treatment. In brief, total RNA in BV2 cells from the following groups (1) negative control, (2) 50 ng/ml LPS, (3) 50 ng/ml LPS+50 *μ*M propofol, (4) 50 *μ*M propofol was extracted by TRIzol (Invitrogen, USA). RNA sequencing analysis was done by Sinotech Genomics (Shenzhen, China). The difference in gene expression between groups was analyzed by the edgeR software package. Differentially expressed genes (DEGs) were defined as follows: fold change larger than 2 or less than 0.5 and have a normalized *p* value less than 0.05. DEGs were enriched by Kyoto Encyclopedia of Genes and Genomes (KEGG). All RNA-seq data in this study have been deposited in the Sequence Read Archive (SRA) repository under accession number PRJNA719268.

### 2.4. Quantitative Real-Time PCR

Total RNA from BV2 cells or primary microglia was isolated using TRIzol reagent (#15596026, Invitrogen, USA) according to the manufacturer's instructions. The RNA concentration and quality were detected by spectrophotometry using NanoDrop™ 2000 (Thermo Scientific, USA). Then, 500 ng of total RNA in 10 *μ*l reaction systems was reversely transcribed by PrimeScript RT Master kit (Cat.# RR036A, Takara Bio Inc., Japan). Real-time qPCR reaction was done with SYBR Green (Bimake, Shanghai, China) using the ViiA7 System (AB Applied Biosystems, USA). The primers used in this study were all acquired from PrimerBank (https://pga.mgh.harvard.edu/primerbank/). *Actb* gene was used as an internal control. Data were analyzed using the *^ΔΔ^*Ct method.

### 2.5. Enzyme-Linked Immunosorbent Assay (ELISA)

The levels of inflammatory cytokines including IL-1beta, IL-6, and TNF-*α* were investigated by commercial ELISA kit from R&D Systems. The detailed kit information was shown as follows: IL-1b (MLB00C), IL-6 (M6000B), and TNF-*α* (MTA00B). The level of each cytokine in the cell culture supernatants was measured according to the manufacturer's instructions. Finally, the concentrations were calculated by using a standard curve, and the data were shown in picogram per milliliter.

### 2.6. Western Blotting

Total cell proteins were prepared from BV2 cells or primary microglia. Protein samples were separated on 8% SDS-polyacrylamide gels and transferred onto polyvinylidene difluoride (PVDF) membranes (Millipore, USA), and nonspecific sites were blocked by incubating PVDF membranes in 5% (*w*/*v*) skim milk in Tris-buffered saline containing Tween 20 (TBS-T). Next, the membranes were washed with TBS-T three times and incubated with primary antibodies against TGM2 (15100-1-AP, Proteintech, dilution 1 : 1,000) or *β*-actin (ab8227, Abcam, dilution 1 : 2,000) at 4°C overnight. On the next day, the membranes were then washed with TBS-T and incubated with goat anti-rabbit IgG (H+L) secondary antibody conjugated with horseradish peroxidase (HRP) for 1 h at room temperature. Finally, the membranes were washed with TBS-T and transferred into Bio-Rad ChemiDoc™ XRS system, and blots were developed using the ECL kit (Millipore, USA).

### 2.7. NF-*κ*B Luciferase Reporter Assay

To measure NF-*κ*B transcriptional activity, BV2 cells or primary microglia were transfected with pGMNF-*κ*B-Luc (GM-022001, Genomeditech). Cotransfection of Renilla luciferase under the control of the SV40 early enhancer/promoter region (pSV40-RL, Promega) was used to normalize for transfection efficiency. After treatment for 48 h, the dual-luciferase reporter assay system (Promega, USA) was used to determine NF-*κ*B reporter activity. All transfections were performed at least two times, in triplicate.

### 2.8. Isolation of Primary Microglia

Primary microglia cultures were prepared from cerebral cortices of newborn C57BL/6J mice. In brief, mice were sacrificed and brains were carefully isolated and dissected in Hanks' balanced salt solution (Life Technologies, USA). Subsequently, forebrains were gently minced, dissociated, resuspended in DMEM/F-12 medium (Gibco, Shanghai, China), and filtered by passing through a 70 mm cell strainer (Sigma, Shanghai, China). Cells were collected by centrifugation at 2,000 rpm at 4°C for 10 minutes. Then, harvested cells were resuspended in DMEM/F-12 containing 10% (*v*/*v*) FBS (Gibco, Shanghai, China) and cultured on poly-D-lysine-coated 75 cm^2^ cell culture flask. After 12-14 days in culture, floating microglia were collected and reseeded into cell culture plates. On the next day, nonadherent cells were removed and cells left were used for subsequent functional experiments.

### 2.9. Statistical Analysis

Data were all shown as the means ± SD. Statistical analysis was conducted with GraphPad Prism 5 (GraphPad Software, San Diego, CA, USA). The two-sided Student *t*-test or one-way ANOVA followed by Student-Newman-Keuls (SNK) test was done to compare data between groups. *p* values less than 0.05 were considered statistically significant.

## 3. Results

### 3.1. TGM2 Is Upregulated in LPS-Primed Microglia and Can Be Downregulated by Propofol

**Figure 1:**
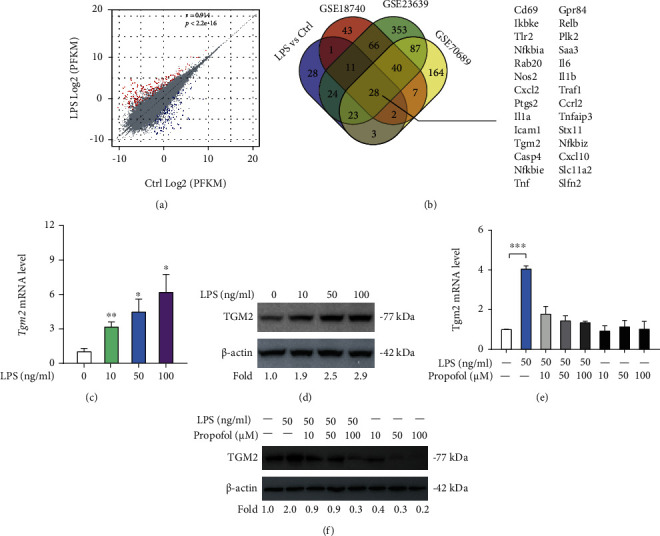
TGM2 is upregulated in LPS-primed microglia and can be downregulated by propofol. (a) Comparison of the RNA sequencing results of control and LPS-primed BV2 cells; scatter plots showed differentially expressed genes (DEGs) related to BV2 microglia activation. (b) Venn diagram showed DEGs related to LPS-induced activation of microglia. (c) Real-time qPCR analysis of *Tgm2* expression in BV2 cells upon treatment with different concentrations of LPS (*n* = 3). (d) Western blotting analysis of TGM2 expression in BV2 cells upon treatment with different concentrations of LPS; *β*-actin was loaded as a control. (e) *Tgm2* expression in LPS-primed BV2 cells upon treatment with different concentrations of propofol was determined by real-time qPCR (*n* = 3). (f) TGM2 expression in LPS-primed BV2 cells upon treatment with different concentrations of propofol was detected by Western blotting. ^∗^*p* < 0.05, ^∗∗^*p* < 0.01, and ^∗∗∗^*p* < 0.001.

To identify genes related to microglia activation, we performed RNA sequencing analysis in BV2 cells with or without LPS stimulation. As a result, 120 upregulated differentially expressed genes (DEGs) and 67 downregulated DEGs were identified (Figure 1(a)). By merging upregulated DEGs from three independent studies (GSE18740, GSE22639, and GSE70689), we noticed that 28 genes (*Cd69*, *Ikbke*, *Tlr2*, *Nfkbia*, *Rab20*, *Nos2*, *Cxcl2*, *Ptgs2*, *Il1a*, *Icam1*, *Tgm2*, *Casp4*, *Nfkbie*, *Tnf*, *Gpr84*, *Relb*, *Plk2*, *Saa3*, *Il6*, *Il1b*, *Traf1*, *Ccrl2*, *Tnfaip3*, *Stx11*, *Nfkbiz*, *Cxcl10*, *Slc11a2*, and *Slfn2*) were commonly upregulated in activated microglia (Figure 1(b)). Actually, most of these genes are directly involved in the inflammatory responses. Notably, TGM2, a multifunctional enzyme that catalyzes the formation of intermolecular isopeptide bonds between glutamine and lysine side chains [22], was selected for detailed investigation. To uncover whether TGM2 is involved in propofol-mediated anti-inflammatory roles in BV2 activation, we first analyzed its mRNA and protein expression upon treatment with different concentrations of LPS (0, 10, 50, and 100 ng/ml) by real-time qPCR and Western blotting, respectively. The result showed that *Tgm2* mRNA level was increased by LPS stimulation in a dose-dependent manner (Figure 1(c)). Consistently, a similar result was also noticed at the protein level (Figure 1(d)). Furthermore, LPS-induced increase of TGM2 expression was significantly downregulated by the addition of propofol (Figures 1(e) and 1(f)), indicating that propofol might target TGM2 to inhibit BV2 microglial activation.

### 3.2. Knockdown or Pharmacological Inhibition of TGM2 Suppresses Activation of BV2 Microglia

**Figure 2:**
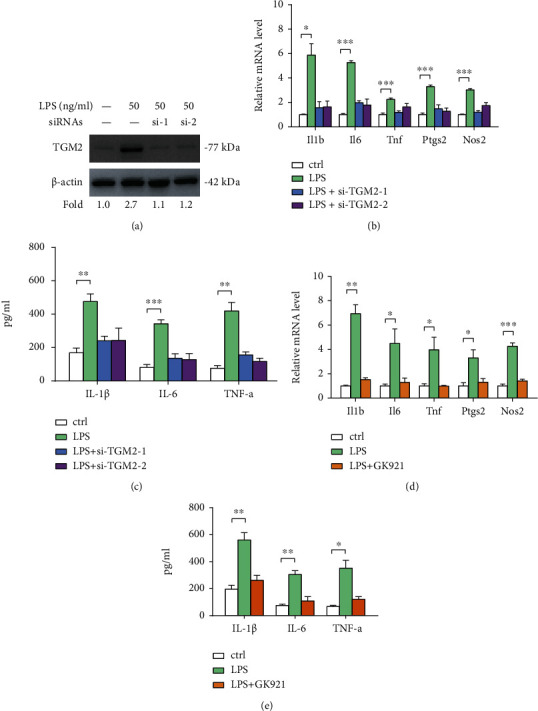
Knockdown or pharmacological inhibition of TGM2 suppresses activation of BV2 microglia. (a) Western blotting analysis of the knockdown efficiency of TGM2 in LPS-primed BV2 cells; *β*-actin was loaded as a control. (b) The effects of TGM2 knockdown on the mRNA expression of inflammatory genes (*Il1b*, *IL6*, *Tnf*, *Ptgs2*, and *Nos2*) in LPS-primed BV2 cells were analyzed by real-time qPCR analysis (*n* = 3). (c) The effects of TGM2 knockdown on the level of inflammatory cytokines (IL-1*β*, IL-6, and TNF-*α*) in conditioned medium from LPS-primed BV2 cells were analyzed by ELISA (*n* = 3). (d) The mRNA expression of inflammatory genes (*Il1b*, *IL6*, *Tnf*, *Ptgs2*, and *Nos2*) in LPS-primed BV2 cells with or without GK921 treatment was analyzed by real-time qPCR analysis (*n* = 3). (e) The level of inflammatory cytokines (IL-1*β*, IL-6, and TNF-*α*) in conditioned medium from LPS-primed BV2 cells with or without GK921 treatment was analyzed by ELISA (*n* = 3). ^∗^*p* < 0.05, ^∗∗^*p* < 0.01, and ^∗∗∗^*p* < 0.001.

To investigate whether TGM2 is involved in microglia activation, we performed loss-of-function studies. Two siRNAs against *Tgm2* in LPS-primed BV2 cells led to a significant decrease in TGM2 protein level (Figure 2(a)). Consistently, LPS-induced upregulation of inflammatory genes (*Il1b*, *IL6*, *Tnf*, *Ptgs2*, and *Nos2*) (Figure 2(b)) and levels of inflammatory cytokines (IL-1*β*, IL-6, and TNF-*α*) (Figure 2(c)) in the cell culture supernatants were also reduced by two siRNAs against *Tgm2*. Next, a small molecule inhibitor GK921 was used to inhibit TGM2 activity in BV2 cells. The result showed that GK921 treatment also suppressed the expression of inflammatory genes and elevated inflammatory cytokines induced by LPS stimulation (Figures 2(d) and 2(e)).

### 3.3. TGM2 Promoted BV2 Activation Independent of Its Transamidase Activity

**Figure 3:**
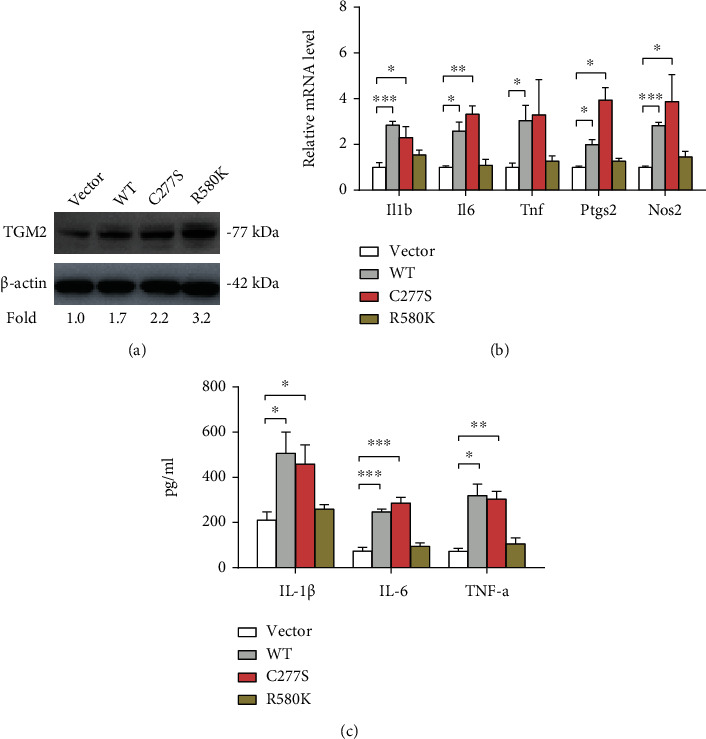
TGM2 promotes BV2 activation independent of its transamidase-activity. (a) Western blotting analysis of the overexpression efficiency of TGM2-WT, TGM2-C277S, and TGM2-R580K in BV2 cells. (b) The effects of TGM2-WT, TGM2-C277S, and TGM2-R580K on the mRNA expression of inflammatory genes (*Il1b*, *IL6*, *Tnf*, *Ptgs2*, and *Nos2*) in BV2 cells were analyzed by real-time qPCR analysis (*n* = 3). (c) The effects of TGM2-WT, TGM2-C277S, and TGM2-R580K on the level of inflammatory cytokines (IL-1*β*, IL-6, and TNF-*α*) in conditioned medium from BV2 cells were analyzed by ELISA (*n* = 3). ^∗^*p* < 0.05, ^∗∗^*p* < 0.01, and ^∗∗∗^*p* < 0.001.

To further confirm the role of TGM2 in the activation of microglia, we overexpressed wide-type TGM2, transamidase-inactive TGM2 (C277S), and GTP-binding-inactive TG2 (R580K) in BV2 cells, respectively. The overexpression efficiency is shown in Figure 3(a). As a result, the WT-TGM2 and the transamidase-inactive TGM2 effectively promoted microglia activation as evidenced by increased expression of inflammatory genes and elevated inflammatory cytokines, whereas the GTP-binding-deficient TGM2 failed to boost BV2 cell activation (Figures 3(b) and 3(c)). These results suggest that the GTP binding activity instead of transamidase activity of TGM2 plays an important role in the induction of microglial activation.

### 3.4. TGM2 Activates NF-*κ*B Signaling to Promote Microglial Activation

**Figure 4:**
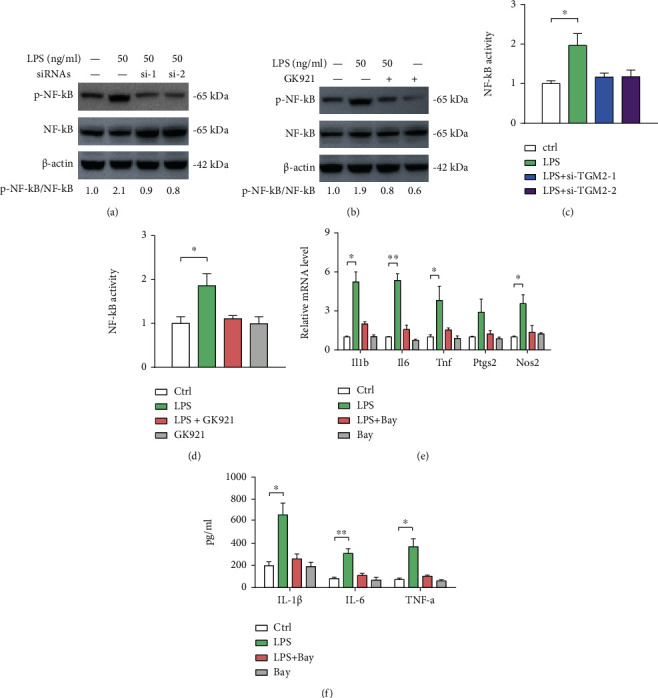
TGM2 activates NF-*κ*B signaling to promote microglial activation. (a) The effects of TGM2 knockdown on the activation of NF-*κ*B signaling in LPS-primed BV2 cells were analyzed by Western blotting. (b) The effects of TGM2 inhibition with GK921 on the activation of NF-*κ*B signaling in LPS-primed BV2 cells were analyzed by Western blotting. (c) The effects of TGM2 knockdown on the NF-*κ*B activity in LPS-primed BV2 cells were analyzed by luciferase reporter assay. (d) The effects of TGM2 inhibition with GK921 on the NF-*κ*B activity in LPS-primed BV2 cells were analyzed by luciferase reporter assay. (e) The effects of Bay11-7082 (10 *μ*M) on the mRNA expression of inflammatory genes (*Il1b*, *IL6*, *Tnf*, *Ptgs2*, and *Nos2*) in LPS-primed BV2 cells were analyzed by real-time qPCR analysis (*n* = 3). (f) The effects of Bay11-7082 (10 *μ*M) on the level of inflammatory cytokines (IL-1*β*, IL-6, and TNF-*α*) in conditioned medium from LPS-primed BV2 cells were analyzed by ELISA (*n* = 3). ^∗^*p* < 0.05 and ^∗∗∗^*p* < 0.001.

The transcription factor NF-*κ*B is an important regulator of inflammation in the central nervous system and can be activated by a variety of stimuli including inflammatory cytokines, calcium influx, and oxidative stress [23]. Given TGM2 is involved in molecular mechanisms underlying microglial activation, we tested the changes of NF-*κ*B signaling in response to TGM2 intervention. In line with previous findings, LPS stimulation led to a significant elevation of p-NF-*κ*B level, which can be inhibited by TGM2 knockdown (Figure 4(a)). Likewise, GK921 treatment also blocked LPS-induced activation of NF-*κ*B signaling (Figure 4(b)). Using luciferase reporter assay, we further confirmed the inhibitory effects of TGM2 knockdown or inhibition on the activation of NF-*κ*B signaling (Figures 4(c) and 4(d)). Additionally, Bay11-7082, a specific inhibitor against NF-*κ*B signaling, significantly suppressed the expression of inflammatory genes and elevated inflammatory cytokines induced by LPS in BV2 cells (Figures 4(e) and 4(f)). Taken together, these findings suggest that TGM2 may activate the NF-*κ*B signaling pathway to induce microglial activation.

### 3.5. Propofol Attenuates NF-*κ*B Signaling to Inhibit Microglial Activation

**Figure 5:**
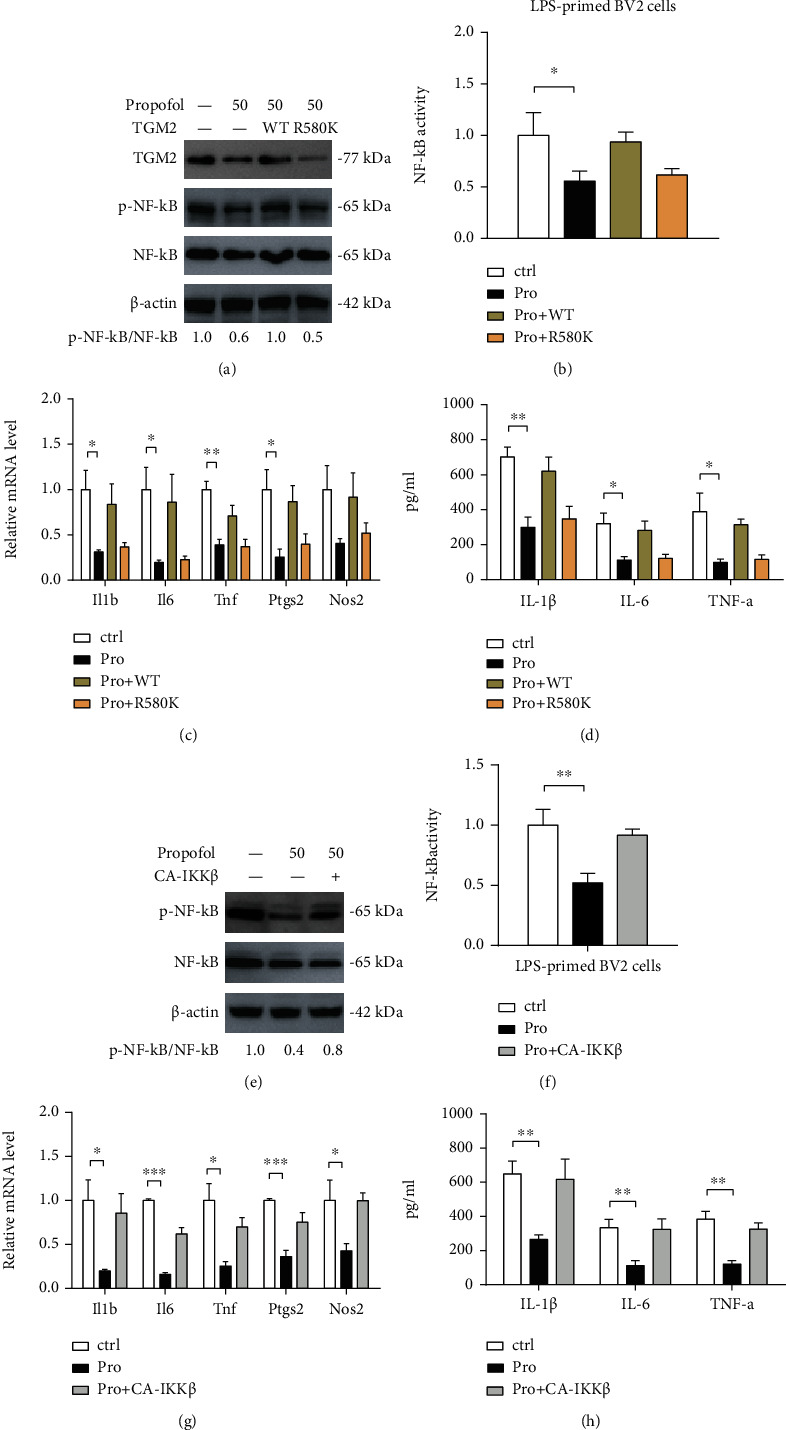
Propofol attenuates NF-*κ*B signaling to inhibit microglial activation. (a) Western blotting analysis of p-NF-*κ*B and NF-*κ*B levels in LPS-primed BV2 cells upon treatment with propofol and/or overexpression of TGM2-WT and TGM2-R580K; *β*-actin was loaded as a control. (b) The effects of propofol and/or TGM2-WT/TGM2-R580K on NF-*κ*B activity in LPS-primed BV2 cells were analyzed by luciferase reporter assay (*n* = 3). (c) The effects of propofol and/or TGM2-WT/TGM2-R580K on the mRNA expression of inflammatory genes (*Il1b*, *IL6*, *Tnf*, *Ptgs2*, and *Nos2*) in LPS-primed BV2 cells were analyzed by real-time qPCR analysis (*n* = 3). (d) The effects of propofol and/or TGM2-WT/TGM2-R580K on the level of inflammatory cytokines (IL-1*β*, IL-6, and TNF-*α*) in conditioned medium from LPS-primed BV2 cells were analyzed by ELISA (*n* = 3). (e) Western blotting analysis of the effect of constitutively active IKK*β* (CA-IKK*β*) on the p-NF-*κ*B and NF-*κ*B levels in LPS-primed BV2 cells with propofol treatment. (f) The effects of CA-IKK*β* on the NF-*κ*B activity in LPS-primed BV2 cells with propofol treatment were analyzed by luciferase reporter assay (*n* = 3). (g) The effects of CA-IKK*β* on the mRNA expression of inflammatory genes (*Il1b*, *IL6*, *Tnf*, *Ptgs2*, and *Nos2*) in LPS-primed BV2 cells with propofol treatment were determined by real-time qPCR analysis (*n* = 3). (h) The effects of CA-IKK*β* on the level of inflammatory cytokines (IL-1*β*, IL-6, and TNF-*α*) in conditioned medium from LPS-primed BV2 cells with propofol treatment were analyzed by ELISA (*n* = 3). *p* < 0.05, ^∗∗^*p* < 0.01, and ^∗∗∗^*p* < 0.001.

Next, we aimed to uncover whether the TGM2-NF-*κ*B axis functionally mediates the propofol-dependent inactivation of microglia. To address this issue, we first overexpressed wide-type TGM2 and GTP-binding-inactive TGM2 (R580A) in LPS-primed BV2 cells upon treatment with propofol (Figure 5(a)). As shown in Figures 5(b)–5(d), propofol significantly inhibited the activity of NF-*κ*B signaling, expression of inflammatory genes (*Il1b*, *IL6*, *Tnf*, *Ptgs2*, and *Nos2*), and inflammatory cytokines (IL-1*β*, IL-6, and TNF-*α*), which can be restored by wide-type TGM2 but not GTP-binding-inactive TGM2. Additionally, we ectopically expressed constitutively active IKK*β* (CA-IKK*β*) in LPS-primed BV2 cells upon treatment with propofol (Figure 5(e)). Expectedly, propofol failed to induce an inhibitory effect on BV2 cells in the presence of constitutive activation of NF-*κ*B signaling as demonstrated by altered NF-*κ*B activation (Figure 5(f)), expression of inflammatory genes (Figure 5(g)), and levels of inflammatory cytokines (Figure 5(h)). Collectively, these findings above indicate that TGM2-mediated activation of NF-*κ*B signaling might be a target of propofol in reducing microglia activation.

### 3.6. Propofol Targets TGM2-NF-*κ*B Signaling in Primary Microglia Activation

**Figure 6:**
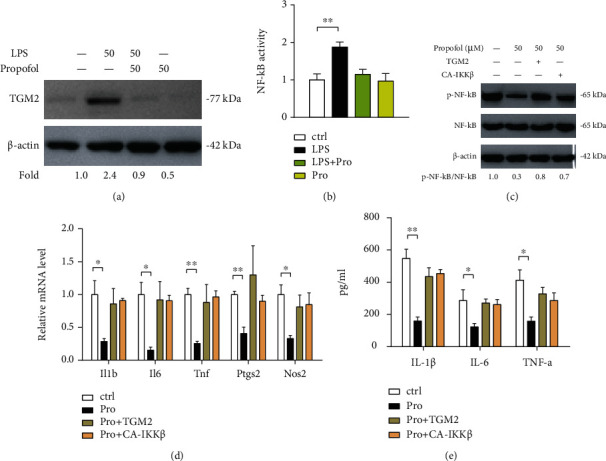
Propofol targets TGM2-NF-*κ*B signaling in primary microglia activation. (a) TGM2 protein expression in primary microglia upon treatment with LPS and/or propofol was determined by Western blotting. (b) The effect of propofol on NF-*κ*B activity in LPS-primed primary microglia was analyzed by luciferase reporter assay (*n* = 3). (c) Western blotting analysis of the effect of constitutively active IKK*β* (CA-IKK*β*) or TGM2 overexpression on the p-NF-*κ*B and NF-*κ*B levels in LPS-primed primary microglia with propofol treatment; *β*-actin was loaded as a control. (d) The effects of constitutively active IKK*β* (CA-IKK*β*) or TGM2 overexpression on the mRNA expression of inflammatory genes (*Il1b*, *IL6*, *Tnf*, *Ptgs2*, and *Nos2*) in LPS-primed primary microglia with propofol treatment were determined by real-time qPCR analysis (*n* = 3). (e) The effects of constitutively active IKK*β* (CA-IKK*β*) or TGM2 overexpression on the level of inflammatory cytokines (IL-1*β*, IL-6, and TNF-*α*) in conditioned medium from LPS-primed primary microglia with propofol treatment were analyzed by ELISA (*n* = 3). ^∗^*p* < 0.05 and ^∗∗^*p* < 0.01.

To further confirm the role of propofol-TGM2-NF-*κ*B signaling in microglial activation, we acquired primary microglia cultures to determine whether propofol has similar anti-inflammatory properties. Indeed, TGM2 protein level was significantly increased upon stimulation with 50 ng/ml LPS, and upregulated TGM2 expression can be drastically downregulated by treatment with 50 *μ*M propofol (Figure 6(a)). Moreover, luciferase reporter assay also revealed that the activity of NF-*κ*B signaling was also attenuated by propofol treatment (Figure 6(b)). To determine the role of propofol and the TGM2-NF-*κ*B axis in primary microglia activation, we overexpressed wide-type TGM2 or CA-IKK*β* in LPS-primed primary microglia with propofol treatment. As shown in Figure 6(c), propofol reduced p-NF-*κ*B protein expression in LPS-primed primary microglia and this inhibitory effect can be restored by ectopic expression of wide-type TGM2 or CA-IKK*β*. Similarly, the expression of proinflammatory genes (*Il1b*, *IL6*, *Tnf*, *Ptgs2*, and *Nos2*) and levels of proinflammatory cytokines (IL-1*β*, IL-6, and TNF-*α*) in cell culture supernatants from primary microglia were also downregulated by treatment with propofol; and consistently, the introduction of wide-type TGM2 or CA-IKK*β* in LPS-primed primary microglia blocked the inhibitory effect of propofol (Figures 6(d) and 6(e)).

## 4. Discussion

Inflammation is associated with diverse CNS diseases such as trauma, stroke, or chronic neurodegenerative disorders. Excessive activation of microglia is profoundly implicated in the neuroinflammation via producing a cascade of inflammatory mediators such as iNOS/NO, IL-1*β*, IL-6, and TNF-*α*, which further result in neuritis, immune response, damage of neurons, and cause cognitive dysfunction [6, 24–26]. Thus, approaches to limit the activation of microglia may reduce inflammatory cascades and will be beneficial to control neuroinflammatory diseases. In the present study, we confirmed the effects of propofol on suppressing microglial activation and identified the TGM2-NF-*κ*B signaling as a mediator in LPS-mediated microglia activation. Propofol can proficiently inhibit the activation of TGM2-NF-*κ*B signaling to prevent microglial activation in both BV2 cells and primary microglia.

The transcription factor NF-*κ*B plays a critical role in the regulation of genes involved in immune response and inflammatory processes and has been associated with many pathological conditions including traumatic brain injury, neuroinflammation, and neurodegeneration [27, 28]. Previously, many studies have documented the effect of LPS on NF-*κ*B signaling in microglia [29–31]. Consistent with these reports, we confirmed that LPS led to a significant increase of p-NF-*κ*B level in BV2 cells and primary microglia, and this effect was largely dependent on the presence of TGM2 as TGM2 knockdown blocked NF-*κ*B activation. Actually, the link between TGM2 and activation of NF-*κ*B has been uncovered in different disease models [32–34]. TGM2 is the most abundant TGM in the CNS and is highly responsive to CNS injury [35]. TGM2 can activate NF-*κ*B signaling via disabling the I*κ*B*α* inhibitor and NF-*κ*B can transcriptionally upregulate TGM2 expression, thus forming a TGM2/NF-*κ*B signaling loop [36]. In the present study, using the site-mutagenesis method, we revealed that the effect of TGM2 on NF-*κ*B activation was dependent on its GTP binding activity instead of its transamidase activity in microglia. In addition to NF-*κ*B signaling, TGM2 can also interact with integrins and growth factor receptors such as epidermal growth factor receptor (EGFR) and induce activation of downstream FAK, Src, and Akt signaling [37]. Moreover, TGM2 can also crosslink its component proteins in the extracellular matrix [38, 39] and play a role in the nucleus via serotonylation [22, 40]. Therefore, we cannot fully exclude the contributions of TGM2-mediated microglial activation from these signaling pathways. Indeed, the neuroprotective roles of propofol have been found by many reports, and many targets of propofol in microglia have been revealed [41–45]. In this study, we for the first time reported that TGM2 is a target of propofol. Moreover, we demonstrated that propofol inhibited the expression of inflammatory genes and release of inflammatory cytokines by attenuating the TGM2-NF-*κ*B signaling, suggesting the crucial role of the propofol-TGM2-NF-*κ*B axis in the process of microglial activation.

Several limitations are also present in the current study. Firstly, how propofol regulates TGM2 expression was not investigated in our study. Secondly, the experiments were performed in BV2 cell line or primary microglia. To strengthen this hypothesis, further data from the mouse model are needed in future studies. In addition, further studies are warranted to clarify the role of NF-*κ*B signaling pathways in the anti-inflammatory activities of propofol in LPS-induced microglial activation.

## 5. Conclusions

The present study highlights the crucial role of propofol in hindering excessive microglial activation and the abnormal release of cytotoxic and inflammatory factors, which would result in serious neuronal damage and further lead to neurodegenerative disorders. Importantly, our findings demonstrate that TGM2-mediated activation of NF-*κ*B acts as a novel molecular target for the neuroprotective effects of propofol. Finally, our study does not imply that propofol is used as a therapeutic drug but rather recommends the use of this anesthetic during surgical procedure, especially for microglia-associated CNS injuries.

## Data Availability

All data generated or analyzed during this study are included in this article.
